# Dissection of Structural Reorganization of Wheat 5B Chromosome Associated With Interspecies Recombination Suppression

**DOI:** 10.3389/fpls.2022.884632

**Published:** 2022-05-04

**Authors:** Elena Salina, Alexander Muterko, Antonina Kiseleva, Zhiyong Liu, Abraham Korol

**Affiliations:** ^1^Institute of Cytology and Genetics, Siberian Branch, Russian Academy of Sciences, Novosibirsk, Russia; ^2^Kurchatov Genomic Center of ICG SB RAS, Novosibirsk, Russia; ^3^Institute of Genetics and Developmental Biology, Chinese Academy of Sciences, Beijing, China; ^4^Institute of Evolution, University of Haifa, Haifa, Israel

**Keywords:** wheat, *dicoccoides*, recombination suppression, chromosome 5B, DNA transposons, LTR retrotransposons, tandem repeats, heterochromatin

## Abstract

Chromosomal rearrangements that lead to recombination suppression can have a significant impact on speciation, and they are also important for breeding. The regions of recombination suppression in wheat chromosome 5B were identified based on comparisons of the 5B map of a cross between the Chinese Spring (CS) variety of hexaploid wheat and CS-5Bdic (genotype CS with 5B substituted with its homologue from tetraploid *Triticum dicoccoides*) with several 5B maps of tetraploid and hexaploid wheat. In total, two regions were selected in which recombination suppression occurred in cross CS × CS-5Bdic when compared with other maps: one on the short arm, 5BS_RS, limited by markers BS00009810/BS00022336, and the second on the long arm, 5BL_RS, between markers Ra_c10633_2155 and BS00087043. The regions marked as 5BS_RS and 5BL_RS, with lengths of 5 Mb and 3.6 Mb, respectively, were mined from the 5B pseudomolecule of CS and compared to the homoeologous regions (7.6 and 3.8 Mb, respectively) of the 5B pseudomolecule of Zavitan (*T. dicoccoides*). It was shown that, in the case of 5BS_RS, the local heterochromatin islands determined by the satellite DNA (119.2) and transposable element arrays, as well as the dissimilarity caused by large insertions/deletions (chromosome rearrangements) between 5BSs *aestivum/dicoccoides*, are likely the key determinants of recombination suppression in the region. Two major and two minor segments with significant loss of similarity were recognized within the 5BL_RS region. It was shown that the loss of similarity, which can lead to suppression of recombination in the 5BL_RS region, is caused by chromosomal rearrangements, driven by the activity of mobile genetic elements (both DNA transposons and long terminal repeat retrotransposons) and their divergence during evolution. It was noted that the regions marked as 5BS_RS and 5BL_RS are associated with chromosomal rearrangements identified earlier by С-banding analysis of intraspecific polymorphism of tetraploid emmer wheat. The revealed divergence in 5BS_RS and 5BL_RS may be a consequence of interspecific hybridization, plant genetic adaptation, or both.

## Introduction

The evolutionary history of the wheat genome has been widely discussed over the last three decades (Goncharov, 2011; Gornicki et al., 2014; El Baidouri et al., 2017; Mirzaghaderi and Mason, 2017; Pont et al., 2019; Li et al., 2022), with genomic changes occurring during both the divergence of its diploid progenitors and the formation of tetraploid and hexaploid forms of wheat and their subsequent evolution. Overall, results suggest that although structural features of the genomes of the diploid species involved in amphiploidization were retained, certain changes also occurred in each round of polyploidization (Jiang and Gill, 1994; Devos et al., 1995; Salina et al., 2006; Marcussen et al., 2014; Jorgensen et al., 2017). The main genomic changes arising during the allopolyploids’ formation were connected to the need for joint “accommodation” of the different genomes in one nucleus and cytogenetic diploidization. The wheat B genome and its progenitor in the diploid species *Aegilops speltoides* are rich in tandem repeats (families pSc119.2, pAs1, GAAGAG, etc.), which are involved in the formation of heterochromatin blocks; in contrast, the A genome is quite lacking in tandem repeat families. This imbalance in the content of tandem repeats in the genomes of diploid progenitors may significantly affect meiotic synchronization in the newly developed amphiploid nucleus. Variability in telomeric heterochromatin and centromeric regions has been seen in wheat-rye hybrids and in their progeny (Fu et al., 2013; Bashir et al., 2018). In the case of the bread wheat B genome, Spelt1 repeats specific to the subtelomeric regions of the diploid progenitor *Ae. speltoides* were lost in this process, which was also experimentally confirmed using newly synthesized amphiploids (Salina et al., 2004). Thus, the imbalance between homoeologous genomes A and B in the content of tandem repeats may decrease as a result of partial deletion of tandem repeats specific to one of the parents during the formation of amphiploids.

The situation with mobile elements is somewhat different. In particular, a study of long terminal repeat (LTR) retroelements in wheats and their progenitors has shown that most transposable elements (TEs) proliferated differentially in diploid progenitors and were then inherited by allopolyploids (Bariah et al., 2020a). Presumably, amphiploidization did not affect the proliferation of retrotransposons (Charles et al., 2008; Salina et al., 2011). However, certain changes in the short interspersed nuclear element (SINE) and non-LTR-retrotransposon families in the second generation of synthetic allohexaploid wheat, and their further active methylation in the third generation, have been reported (Ben-David et al., 2013). Thus, the reorganization of mobile elements at stages following the formation of the first amphiploids warrants further analysis.

One of the most important areas in allopolyploid research is determining the activity of the genes once they undergo duplication. Various interacting mechanisms can partially switch off genes *via* methylation or mutations of duplicated genes (Feldman and Levy, 2012). The consequences of such processes are of paramount importance in allopolyploid organisms. Thus, some 45S RNA genes are inactivated by methylation in chromosomes of one of the species constituting the allopolyploid genome in order to maintain the necessary number of 45S RNA in the cells (Shcherban et al., 2008). The *Ph1* locus emerged in allopolyploid species to prohibit homoeologous pairing in allopolyploid meiosis. Candidates for the *Ph1* gene, as well as the molecular mechanisms underlying its effect, have been discussed (Griffiths et al., 2006; Bhullar et al., 2014; Fan et al., 2021). The formation of an active *Ph1* locus on chromosome 5B also reflects the consequences of interactions between homoeologous gene loci that include activation of genes on one chromosome accompanied by silencing of their orthologues on the homoeologous chromosomes. Chromosome 5B of common wheat is known for the fact that its reorganization throughout the course of evolution led to the emergence of the *Ph* locus, which then contributed to stabilization of the genome of the tetraploid wild emmer wheat *Triticum dicoccoides* and other widespread allopolyploid *Triticum* species. The emergence of *T. dicoccoides*, a wild wheat species of the emmer group, was accompanied by species-specific translocation involving the arms of chromosomes 4AL, 5AL, and 7BS, which was then transmitted to tetraploid and hexaploid species of the emmer group, including cultivated wheat (Naranjo et al., 1987; Devos et al., 1995; Nelson et al., 1995). Chromosome 5B was not involved in large translocation rearrangements during the allopolyploidization.

At the same time, the use of the LTR retrotransposon family in wheat as a genetic marker allowed to identify large-scale genomic rearrangements between the reference chromosome 5B sequences of wild emmer and bread wheat. Six cases of large-scale rearrangements were identified, including 4 cases of long deletions in bread wheat, the introduction of a new DNA fragment, and a single example of copy number variation of a long tandem repeat in chromosome 5B (Bariah et al., 2020b).

A high level of intraspecific polymorphism for chromosome 5B has been noted for the allopolyploid emmer wheat group (*T. dicoccoides, Triticum dicoccum, Triticum aestivum*). Translocations between the long arm of chromosome 7A and the short arm of chromosome 5B were detected in *T. dicoccoides* and *T. dicoccum* (Badaeva et al., 2015). There is a high level of population-specific and region-specific polymorphisms in the distribution of C-bands on the long arm of chromosome 5B in emmer wheat (Badaeva et al., 2015). A group of cultivars of the hexaploid wheat *T. aestivum* that carry 5BS:7BS and 5BL:7BL translocations resulting from intragenomic rearrangements have been bred and are still successfully grown in Western Europe (Badaeva et al., 2007). Thus, chromosome 5B has definite potential for reorganization, resulting in the emergence of intraspecific polymorphisms in common wheat that are utilized in breeding programs.

One significant consequence of intra- or interspecies chromosome rearrangements is the suppression of recombination in hybrids obtained from crossing forms that differ in such rearrangements. Chromosomal rearrangements that lead to recombination suppression can have a significant impact on genetic divergence and even speciation, but are also important for practical breeding.

In the present work, we evaluated the key points of chromosome 5B reorganization throughout evolution by identifying regions of recombination suppresssion in interspecific crosses. Sequence analysis of 5B pseudomolecules of *T. aestivum* (2*n* = 6x) and *T. dicoccoides* (2*n* = 4x) in the studied regions on 5B allowed us to identify possible determinants of recombination suppression.

## Materials and Methods

### Genetic Linkage Maps

In this work, we used the genetic linkage map of CS and a disomic substitution line in which the CS 5B chromosome is replaced with the *T. dicoccoides* 5B chromosome (CS-5Bdic; Salina et al., 2018). Tetraploid wheat maps developed with populations from a cross of *T. durum* and *T. dicoccoides* were also used. The first map, based on a Svevo × Zv recombinant inbred line (RIL) population, was constructed and published by Avni et al. (2014), and the second map was based on a Langdon × Hermon (TZ-2) RIL population, which was genotyping previously (Wang et al., 2018). Both maps were developed using MultiPoint-UltraDense software. The hexaploid wheat map was constructed using the same tools, with the genotyping data of the Chara × Glenlea doubled haploid population kindly provided by E. Akhunov (Kansas State University) and previously published (Wang et al., 2014). The information about the mapping populations used in this study is summarized in Supplementary Table S1.

### Comparative Analysis of Genetic Maps for Chromosome 5B

To compare marker order between genetic linkage maps, The Genetic Map Comparator (Holtz et al., 2017) and BioMercator v4 (Sosnowski et al., 2012) were used. To identify chromosomal regions showing considerable recombination suppression, we compared distances between corresponding markers in CS × CS-5Bdic and the tetraploid and hexaploid wheat maps mentioned above. The regions in CS × CS-5Bdic where the distances between the markers were reduced by more than 3–3.5 times compared to the tetraploid and hexaploid wheat were selected for the following analysis. Information about markers flanking these regions of suppression is presented in Supplementary Table S2.

The recombination regions mapped to deletion bins were performed based on CS × CS-5Bdic genetic mapping data using SNP, SSR, ISBP (insertion site-based polymorphism) markers and localization SSR, ISBP, and SNP markers in bins (Gadaleta et al., 2014; Salina et al., 2018).

### Comparative DNA Analysis for Target Regions of Chromosome 5B

Regions of chromosome 5B where recombination was suppressed according to the results of the genetic analysis were extracted from the reference pseudomolecules of *T. aestivum* cv. CS (IWGSC RefSeq v2.1)^1^ and *T. dicoccoides* cv. Zv (WEWSeq v.2.0)^2^ based on localization of flanking DNA markers. Genome assembly of CS line 42 (CS42, PRJNA392179) was retrieved from the NCBI server.^3^ A sequence similarity search against pseudomolecules of *T. aestivum* varieties Arina (Switzerland), Jagger (United States), Julius (Germany), and Landmark (Canada) was carried out on the IPK server.^4^ Multiple sequence alignment was carried out using MAFFT 7.311 software (Katoh and Standley, 2013).

Tandem repeats were identified with Tandem Repeats Finder software (Benson, 1999) using the default settings. All nested repeats were grouped. Groups separated by less than 100 bp were merged into the same cluster. The obtained clusters containing tandemly repeated DNA sequences were filtered by length ≥ 1 kb. For each cluster containing repeats of the pSc119.2 satDNA family, the sequences of the repeated motif were aligned, and a consensus sequence was obtained.

To calculate the map coverage, local DNA sequence alignments were generated using NUCmer, included in MUMmer (Kurtz et al., 2004) with the following parameters: “-mum -b 150 -c 600 -g 30 -l 24,” and local alignments with ≥80% similarity to the reference sequence were studied.

DNA-sequence alignments for the collinearity analysis were obtained with BLASTn (Zhang et al., 2000; Camacho et al., 2009) and filtered by length (≥3,000 bp) and identity (≥75%). The assemblies containing non-overlapping fragments ordered on the same strand were calculated, and the most representative assembly was taken for downstream analysis.

TEs were recognized in RepeatModeler (v2.0.2, Smit et al., 2008) and RepeatMasker (v4.1.2-p1, Smit et al., 2013) using the RepBase database (RepBaseRepeatMaskerEdition- 20181026, Bao et al., 2015).

Dot plots of 5BS_RS and 5BL_RS regions were calculated in Gepard (v.1.40, Krumsiek et al., 2007) using a word length of 30 bp. Circular data visualization was performed in Circos (Krzywinski et al., 2009).

### DNA Shape Analysis

Modeling of predicted DNA helical paths and calculations of DNA shape features were performed according to previously published methods (Muterko, 2017). The curvature distribution was analyzed in a sliding window of 40 bp with a 1-bp step. DFT was performed with a sampling frequency of 1,024. Three-dimensional DNA structures were visualized by the PyMOL Molecular Graphics System, version 1.7.2 Schrödinger, LLC.

### Evaluation of LTR-Retrotransposon Divergence

Putative LTR retrotransposons with LTR–LTR divergence ≥60% were predicted with LTRharvest (Ellinghaus et al., 2008), and elements with a LTR length ≥ 180 bp were selected. LTR of each element was aligned, and free-end gaps of 150-bp fragments were used in computing divergence. LTR TE divergence was calculated from LTR–LTR mismatches, assuming InDels as single evolutionary events. The mean LTR–LTR divergence (age) is the local maximum of the Gamma distribution, with the shape and scale parameters obtained from the negative binomial distribution fitted to the LTR–LTR mismatch distribution. Furthermore, to evaluate the fractions of LTR–LTR divergences (ages) and improve the fit to the LTR–LTR mismatch distribution, the mixture of Poisson distributions was resolved with the use of the expectation–maximization algorithm. Goodness of fit was evaluated using the coefficient of determination (*r*^2^). LTR TE carrying a number of mismatches equal to or less than the mean LTR divergence was considered young, while all others were considered old. The over- and under-representation of young LTR elements was evaluated in a sliding window of 10 elements with a 1 element step, using hypergeometric test.

## Results

### Comparative Analysis of Chromosome 5B Genetic Maps

To identify chromosome 5B rearrangements that have occurred during evolution, we compared chromosome 5B recombination frequency in hybridizations of tetraploid (*T. durum* × *T. dicoccoides*) and hexaploid (*T. aestivum* × *T. aestivum*) wheat and in hybrids of hexaploid wheat cv. Chinese Spring (CS) and a disomic substitution line in which CS chromosome 5B has been replaced with *T. dicoccoides* chromosome 5B (CS-5Bdic). Only populations genotyped with Illumina Infinium arrays were used in this work (Supplementary Table S1). All of the maps were constructed using MultiPoint-UltraDense software.

**Figure 1:**
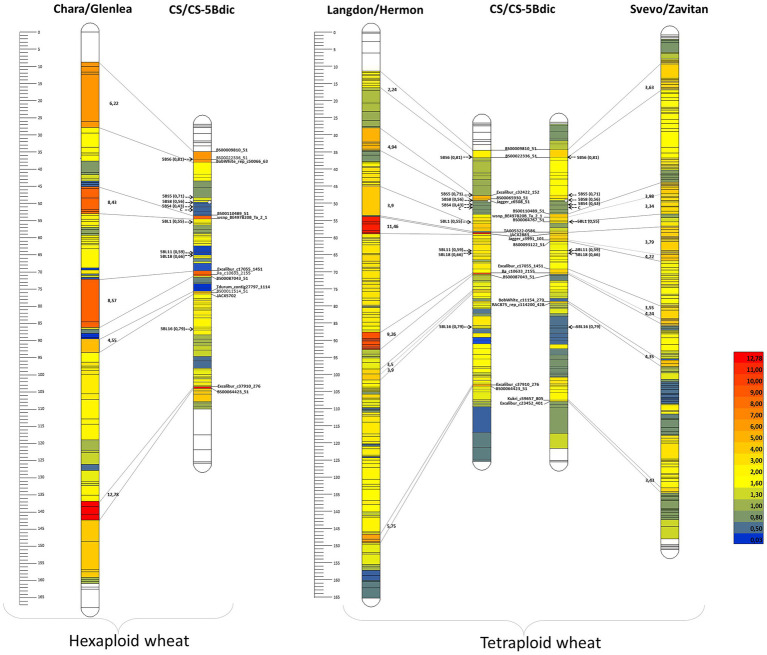
Comparison of CS × CS-5Bdic RIL map with maps of three populations: RIL *T. durum* х *T. dicoccoides* (Svevo/Zavitan); *T. durum* х *T. dicoccoides* (Langdon/Hermon); double haploid population from crossing common wheat Chara х Glenlea. The level of suppression compared to CS × CS-5Bdic is displayed on a color scale; arrows indicate centromeres and breaking points of bin; distances on ruler are presented in cM; lines are connected to the regions with suppression of more than 3–4 times. The deletion bins are points 5BL16, 5BL18, 5BL11, 5BL1, 5BS4, 5BS8, 5BS5, and 5BS (Salina et al., 2018).

A comparison of the maps demonstrated that the used markers were arranged in the same order (Figure 1). Although the total number of markers in the Chara × Glenlea and Svevo × Zavitan (Zv) maps compared to that of CS × CS-5Bdic was larger, the number of skeletal markers (markers representing groups of cosegregating markers occupying separate positions on the constructed linkage map) varied to a lesser degree. A comparative analysis of the length of the 5B maps and the distances between corresponding markers in the studied tetraploid and hexaploid populations relative to CS × CS-5Bdic showed that the latter was 1.5- to 1.7-fold shorter.

The regions with suppressed recombination are evident on the 5B map of CS × CS-5Bdic (Figure 1). In these regions, the distances between the markers are reduced by more than 3–3.5 times, as compared to the maps constructed for tetraploid (Svevo × Zavitan and Langdon × Hermon) and hexaploid (Chara × Glenlea) wheats and exceed the whole map reduction coefficient (1.5–1.7) by more than twofold. Two regions of recombination suppression on the short arms (between markers BS00009810 and BS00022336) and long arms (between markers Ra_c10633_2155 and BS00087043) are observed in all comparisons of intra- versus interspecies chromosome maps (all markers cosegregating with these basic markers are summarized in Supplementary Table S2).

Recombination suppression is presumably associated with the fact that hybridization in the case of CS × CS-5Bdic involved chromosomes 5B of common wheat and the wild species *Triticum dicoccoides*, which may have discrepancies along the 5B sequence. In addition, heterochromatin and possibly associated epigenetic regulation may be involved in recombination suppression (Termolino et al., 2016) and divergence of sequences of related wheat species. For a better understanding of the potential causes of recombination suppression in specific regions of chromosome 5B, the 5B pseudomolecules of *Triticum* species need to be compared.

### Comparative Analysis of *Triticum aestivum* and *Triticum dicoccoides* Chromosome 5B Regions Associated With Recombination Suppression in Interspecific Cross

To define the possible determinants of recombination suppression, we analyzed the primary structure of the two chromosome 5B regions of *T. aestivum* and *T. dicoccoides*: (a) 5BS_RS, between markers BS00009810 (same position as BS00083715: 10489937–10,490,037 bp (CS chr5B pseudomolecule), 9,941,766–9,941,866 bp (Zv chr5B pseudomolecule)) and BS00022336 (15585936–15,586,036 bp (CS), 17,514,968–17,515,068 (Zv)); and (b) 5BL_RS, between markers Ra_c10633_2155 [same position as Tdurum_contig 25513_123: 549917016–549,917,116 bp (CS), 561,877,394–561,877,494 bp (Zv)] and BS00087043 [553533144–553,533,244 bp (CS), 565,677,935–565,678,035 bp (Zv)]. The regions of recombination suppression on the short and long arms, with lengths of 5 Mb and 3.6 Mb, respectively, were mined from the 5B pseudomolecule of CS and compared to the homoeologous regions (7.6 and 3.8 Mb, respectively) of the 5B pseudomolecule of *T. dicoccoides* Zv.

### 5BS_RS Regions

**Figure 2:**
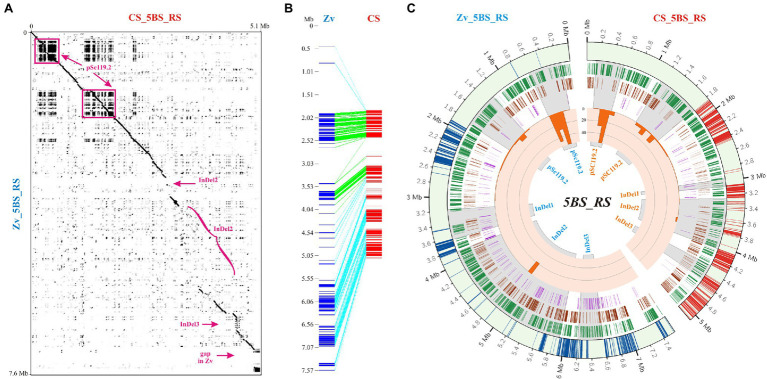
Comparative analysis of the 5BS_RS region in Chinese Spring (CS) and Zavitan (Zv). **(A)** Dot plot of the 5BS_RS DNA sequences from the CS and Zv pseudomolecules. Localization of highly diverged regions containing insertions/deletions (InDels) and a 300-kb gap in Zv is indicated. **(B)** Collinearity between the 5BS_RS regions of CS and Zv assembled from fragments of >3 kb in length and > 75% identity between species. **(C)** Distributions of TEs and satDNA for the 5BS_RS regions of CS and Zv. Four datasets are represented in the circular diagram, from outside to inside: distribution of LTR retroelements (track 1, green highlights), DNA transposons (track 2, brown highlights), unknown TEs (track 3, purple highlights); the fourth track (inside – orange) includes a histogram of the satDNA coverage distribution.

Comparison of the 5BS_RS regions of CS and Zv revealed two areas of tandem repeat sequences and several areas that had either very low similarity or did not map to CS chromosomes at all (Figure 2A). The latter included different chromosomal rearrangements, with a predominance of insertions/deletions (InDels; Figure 2B). This also followed from the difference in length of the 5BS_RS region on pseudomolecules of CS and Zv. In particular, the 5BS_RS of Zv includes around 2 Mb of the DNA sequences, which did not map to the collinear segment of the CS chromosome, suggesting that they are the major contributors to recombination suppression in the 5BS_RS region through loss of similarity.

Extended arrays of tandemly repeated DNA sequences (satellite DNA [satDNA]) were localized to the beginning of the 5BS_RS region. A comparison of fractions containing clusters of tandem repeats with repetitive elements of different lengths, as well as an evaluation of the total length of repetitive sequences in each fraction, showed that repeated sequences within 5BS_RS are mainly represented by clusters of a 118-bp tandem unit or its dimer (235–236 bp) or trimer (353–354 bp). Primary structure analysis has shown that this highly repetitive sequence belongs to the pSc119.2 satDNA family, which is widely distributed within the *Triticeae*, as well as in some *Aveneae* species, and forms a large and evolutionarily old component of the genome (Contento et al., 2005). For a more detailed investigation of distribution and genetic diversity, the arrays of 118-bp tandem repeats of the pSc119.2 family were clustered and filtered, as described in Experimental Procedures, and a consensus sequence was deduced for each cluster. The analysis of pSc119.2 distribution identified three large pools (sized ~500, 600, and 90 kb) located on half of the 5BS_RS region (Figures 2A,C; Supplementary Figure S1). A total of 113 clusters of pSc119.2 were identified for CS and 82 for Zv (Supplementary Figure S1), even though both provided similar coverage (Figure 2C).

With the exclusion of single clusters, the 118-bp motif was highly conserved in each pool (identity of 86–93%) and between pools (94%) for both CS and Zv. Similarity between consensus DNA sequences of pSc119.2 pools of CS and Zv was 95–97%. Multiple sequence alignment found that pool 2 in CS contains an array of inverted clusters (Supplementary Figure S1). This is an important feature distinguishing pool 2 of the pSc119.2 clusters in CS and Zv. The total length of this inverted region was more than 100 kb. Furthermore, the chromosome region within pool 3 in both CS and Zv was inverted compared to pool 1 (as well as pool 2 of Zv and a part of pool 2 from CS). Although the presence of inverted repeats can lead to their instability in the genome, high-level identity in both the position and primary structure was revealed for the studied pSc119.2 repeats between wild tetraploid and cultivar hexaploid wheat (Supplementary Figures S1, S2).

**Figure 3:**
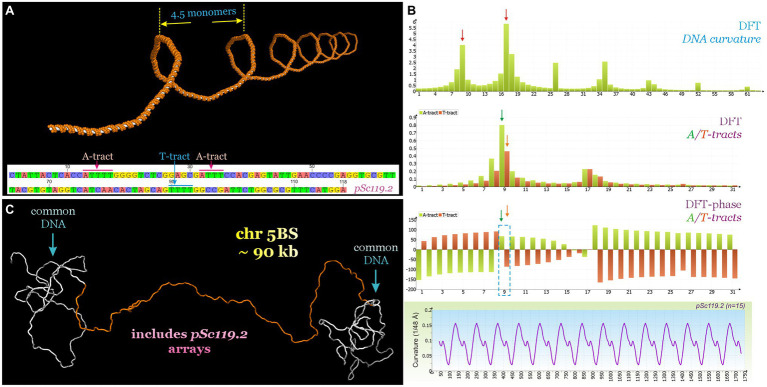
**(A)** Predicted DNA helical path of the pSc119.2 monomer repeated 30 times. **(B)** Parts of DFT power spectra (sampling frequency is 1,024) of curvature and A/T-tract distributions, DFT-phase spectrum of A/T-tract distributions as well as curvature distribution of pSc119.2 array. **(C)** Predicted DNA trajectory of fragment of 5BS_RS region (704–1,012 kb) containing 90-kb pSc119.2 arrays (690–780 kb).

Such stability of the tandem repeat areas is presumably determined by the tertiary structure of the DNA. The DNA sequence of pSc119.2 includes three A-tracts (AnTn, *n* > 3). A-tracts are known as key determinants of the local bends and global curvature of the DNA molecule (Marini et al., 1982; Koo et al., 1986). This global DNA bending can increase significantly when A-tracts are repeated in phase with the helical screw (Marini et al., 1982; Koo et al., 1986; Koo and Crothers, 1988), i.e., are localized in a molecule such that the intrinsic curvature progressively increases in a certain direction. The tandemly repeated sequence containing A-tracts can easily satisfy such conditions. To evaluate the contribution of this satDNA to molecular bending, the predicted DNA helical path for the 118-bp motif of pSc119.2 was calculated and the distribution of the DNA curvature was analyzed (Figure 3A). We found that assuming a DNA helical period of 10 bp, the 118–120-bp periodicity of the pSc119.2 motif is in phase with the DNA helical screw. Two consecutive A-tracts within the analyzed pSc119.2 motif also alternated in phase with the DNA helix (20-bp distance), leading to an increase in the macroscopic DNA curvature. Since A-tracts and T-tracts produce bends in opposite directions, we suggest that those alternating at the same frequency and in phase with the helical screw should mutually compensate for each other. However, analysis of the discrete Fourier transform (DFT)-phase spectra of A- and T-tract alternations showed a phase shift of up to 150° (Figure 3B). Thus, T-tracts alternating out of phase with A-tracts, but producing a bend in the opposite direction, will increase the macroscopic curvature of the DNA molecule. This prediction also follows from the DFT power spectrum of the curvature distribution, where the two detected major frequency bins were 9 and 17, corresponding to 118- and 60-bp periodicity, respectively. One of the important features of regularity is the formation of extended homogeneous rigid structures. In the considered case, this structure is almost a straight helix with a 4.5-monomer period. To demonstrate this, the three-dimensional DNA helical path was inferred for a 90-kb fragment of pSc119.2 arrays (pool 1) flanking the common DNA sequences (Figure 3C). The assumption of involving of satDNA in heterochromatin formation was supported with the use of publicly available for CS the distribution of the chromatin states, determined with a multivariate hidden Markov model for different epigenetic marks (Li et al., 2019). It was shown that the pSc119.2 repeat-containing regions within 5BS_RS are associated with the E13 chromatin state, whose signature is the modified histone H3K9me2 (Supplementary Figure S3), found in constitutive heterochromatin of many eukaryotes. Furthermore, the DNase I profiling (Li et al., 2019) also indicates the low chromatin accessibility within these regions of 5BS_RS.

Although the presence of pSc119.2 tandem repeats providing denser DNA packaging in heterochromatin areas does not explain the observed suppression of recombination, the presence of constitutive heterochromatin can enhance the influence of adjacent regions that are associated with the recombination suppression.

As noted above, a region with low similarity between the homologues can significantly contribute to recombination suppression in heterozygotes. Comparison of the 5BS_RS regions in CS and Zv revealed three large, highly diverged consecutive segments containing InDel-1: ~0.11 and 0.46 Mb, InDel-2: 0.77 and 2.1 Mb, and InDel-3: 0.25 and 0.38 Mb on CS and Zv pseudomolecules, respectively (Figure 2A). Detailed analysis of these regions characterized predominant insertions of TEs (Figure 2C). InDel-1 contained a 0.39-Mb insertion in CS and 0.28-Mb insertion in Zv. Within InDel-2, a total insertion length of 1.6 Mb was detected for Zv and 0.4 Mb for CS. InDel-3 included insertion of repeated DNA sequences in CS, most of which were nested. Zv also contained repeats in this region; however, they were not similar to those in CS and differed in lengths.

**Figure 4:**
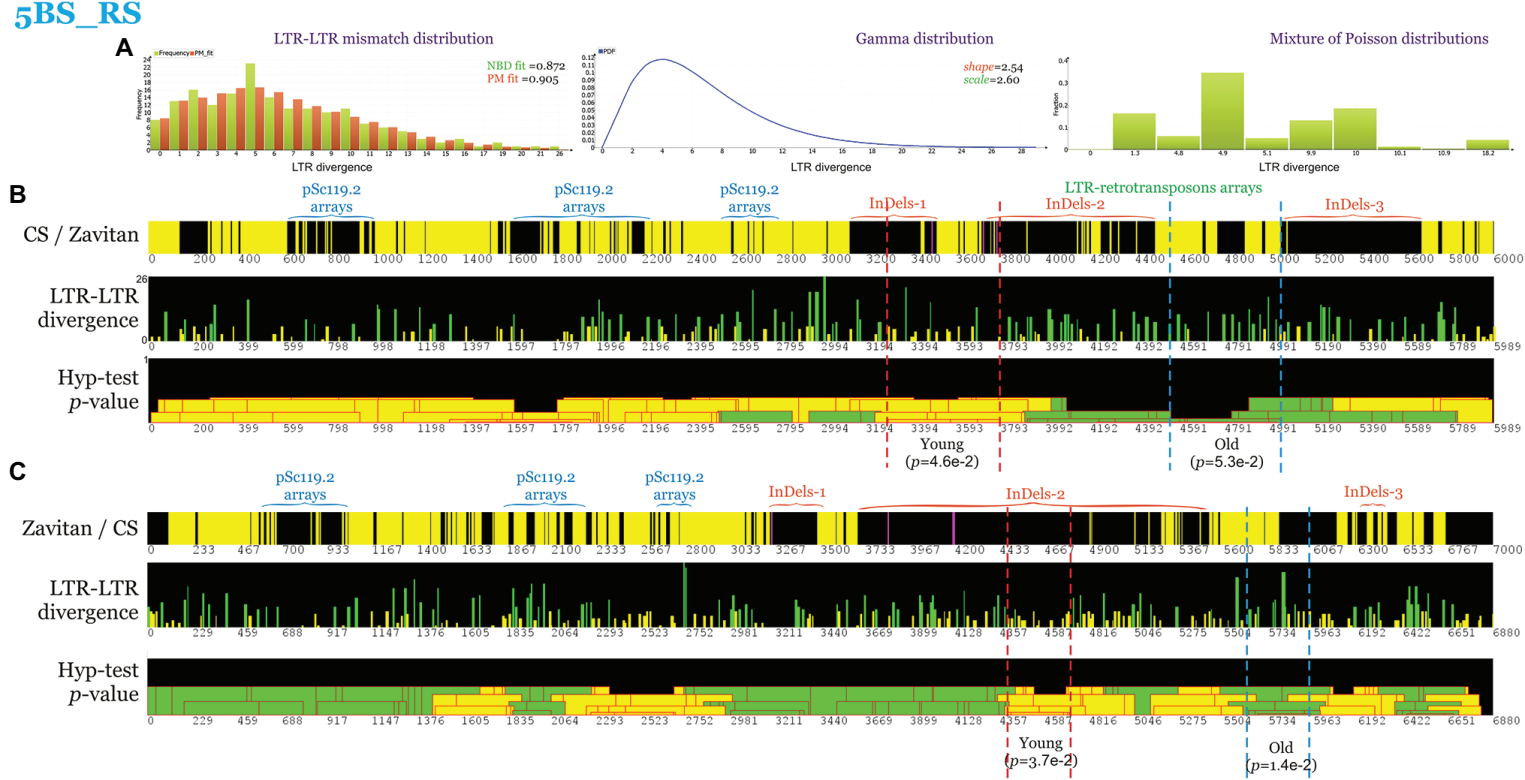
Coverage maps and LTR-LTR divergence distribution within 5BS_RS. Positions are indicated in kb. **(A)** LTR-LTR mismatch distribution and mixture of Poisson distributions (PM) fit. Goodness of fit to negative binomial distribution and PM are indicated. Gamma distribution with the shape and scale parameters obtained from NBD. Mixture of Poisson distributions (abscissa—lambda values, ordinate—proportions). **(B)** Coverage maps and LTR–LTR divergence distribution of CS 5BS_RS region. LTR-TEs with 5 or less LTR-LTR mismatches were considered young (yellow, < 1.28 million years), while other—old (green, more than 1.28 million years). The *p*-values of the over- and under-representation of young LTR-elements are indicated. **(C)** Coverage maps and LTR-LTR divergence of Zavitan 5BS_RS region. Yellow and purple colors indicate the forward and reverse DNA strand, respectively, while black color indicates regions with similarity less than 80% or gaps.

The mobile genetic elements identified within the first and second InDel regions were predominantly the TEs of the LTR order of the class retrotransposons, superfamily Gypsy, and Copia (Supplementary Figure S2). However, it is not clear when during the evolution of polyploid wheat these chromosomal rearrangements occurred. To shed light on this problem, the LTR-retrotransposon age distribution was analyzed along the 5BS_RS chromosome region, and the over- and under-representation of “young” LTR elements was evaluated within the segments where rearrangements in CS and Zv are localized (Figure 4). Analysis of LTR–LTR mismatch distribution in CS found the prevalent fraction of LTR divergence to be five mismatches (Figure 4A). Thus, the elements carrying five or less LTR–LTR mismatches were considered “young,” whereas longer ones were regarded as “old.” Assuming a substitution rate of 1.3 × 10^−8^ mutations per site per year for the plant LTR retrotransposons (Ma and Bennetzen, 2004), this age threshold corresponds to insertion of the LTR retrotransposons before and after 1.3 million years ago for “young” and “old” retrotransposons, respectively.

Statistically significant over-representation of “young” LTR elements (*p* = 0.046 and 0.037, respectively) was shown for two regions (300 and 500 kb), including InDel-1 in CS and the proximal part of InDel-2 in Zv (Figure 4B). The under-representation of young LTR elements was shown for a 1-Mb region between InDel-2 and InDel-3 in CS and Zv (Figures 4B,C).

Thus, given a wild emmer emergence time of about 0.5 MYA (Feldman and Levy, 2012) and according to the estimated time of movement of mobile elements, the rearrangements observed in the studied regions of chromosome 5BS took place in diploid progenitors of allopolyploid wheat and accompanied the processes of evolution and the formation of allopolyploids.

### 5BL_RS Regions

**Figure 5:**
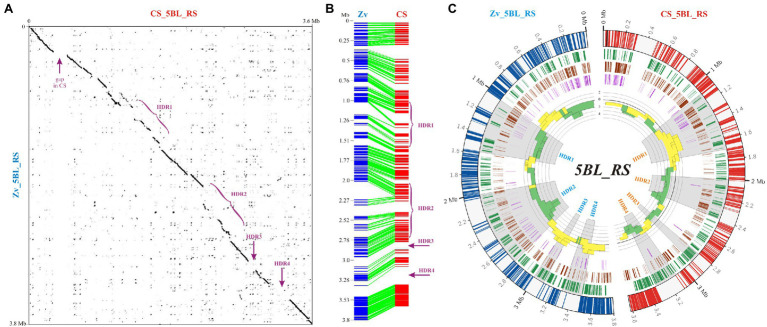
Comparative analysis of the 5BL_RS region in Chinese Spring (CS) and Zavitan (Zv). **(A)** Dot plot of the 5BL_RS DNA sequences from CS and Zv pseudomolecules. Localization of highly diverged regions and 200-kb gap in CS is indicated. **(B)** Collinearity between the 5BL_RS regions of CS and Zv assembled from fragments of >3 kb in length and > 75% identity between species. **(C)** Distributions of TEs and LTR-(retrotransposon?) age analysis for 5BL_RS regions of CS and Zv. Four datasets are represented in the circular diagram, from outside to inside: distribution of LTR retroelements (track 1, green highlights), DNA transposons (track 2, brown highlights), unknown TEs (track 3, purple highlights); the fourth track includes a histogram of the -log10 of *p*-values of the hypergeometric test for the over-representation of “young” (outside – yellow) and “old” (inside—green) LTR elements.

Despite the apparent similarity of the 5BL_RS segment between the CS and Zv genome assemblies, our analysis revealed several highly diverged regions (HDRs, Figure 5A). This loss of similarity was mainly caused by insertions and deletions (InDels) as it followed from the different lengths of 5BL_RS in CS and Zv pseudomolecules (3,617 and 3,802 Mb, respectively) and displacements in the distribution of collinear segments (Figure 4B). In particular, from 0.3 to 2 Mb (Figure 5B), the displacement of collinear segments implied insertions in CS (or deletions in Zv), and from 2 Mb on, insertions in Zv (or deletions in CS). DNA sequence analysis of these regions revealed that in the first case, the initial offset of collinear segments is caused by the prolonged gap in CS (0.3–0.48 Mb), while all following displacements in this area, beginning from 1.16 Mb, are caused by insertions of additional sequences in the CS genome (Figure 5B). We conclude that in the second case, insertions of additional DNA sequences in the Zv genome beginning from a position of 2 Mb occurred at least twice, leading to the displacement of collinear segments within the alignment in the opposite direction.

Overall, two major and two minor segments with significant loss of similarity could be recognized within the 5BL_RS region (HDR1–4, Figure 5A). The first (HDR1) is localized proximal to the Tdurum_contig25513_123 marker (1–1.57 Mb) and includes around 550 kb on the 5B pseudomolecules of CS and Zv. The second (HDR2) begins near the center of 5BL_RS and expands to the BS00087043_51 marker (2–2.7 Mb). It encompasses almost 350 kb in CS and is twice as long in Zv (close to 700 kb), assuming insertions with a total length of 350 kb within the Zv genome. Although both of these segments are enriched with TEs, different types of TEs are not equally represented. In particular, in HDR1, the DNA transposons are prevalent over LTR retrotransposons (Figure 5C), and among them, elements of the DNA/CMC-EnSpm family are represented almost exclusively. Two short (around 0.1 and 0.2 Mb) distal segments (HDR3 and HDR4, respectively) with a complete absence of similarity were enriched in LTR retrotransposons (Figure 5C).

To investigate the possible reasons for divergence of HDRs within 5BL_RS, analysis of the LTR age distribution was carried out exactly as described above for 5BS_RS. The results obtained for Zv showed that in the HDRs of 5BL_RS, where LTR elements are few and DNA transposons are prevalent (HDR1 and HDR2), there is a statistically significant over-representation of the “old” LTR retrotransposons, whereas in the HDRs where “young” LTR elements are over-represented (HDR3 and HDR4), the DNA transposons are less frequent (Figure 5C, left side). Except for HDR2, opposite results were obtained for CS (Figure 5C, right side). In fact, this opposition is in good accordance with the occurrence of HDRs, which allows us to explain the suppression of recombination on 5BL_RS for homologous chromosomes of the considered wheat species. In particular, the over-representation of the “young” retroelements in HDR1 of CS agrees with the insertion of additional DNA sequences in this region (rather than their selective deletion in Zv) and hence contributes to the lowering of similarity with a collinear chromosomal segment in Zv for which the “old” LTR retrotransposons are over-represented on this region. Similarly, over-representation of the “young” LTR elements in HDR3 and HDR4 of Zv corresponds to over-representation of the “old” LTR elements in the collinear regions of CS (Figure 5C).

Based on these data, we suggest that the loss of similarity, which can lead to recombination suppression in the 5BL_RS region, is caused by chromosomal rearrangements, driven by the activity of mobile genetic elements (both DNA transposons and LTR retrotransposons) and their divergence during evolution.

It should be noted that in CS, HDR2 includes a scaffold of around 400 kb (scaffold2564–2), which is flanked by a long span of simple tandem repeats (STRs) of the same type: (TCTTCT)n, *n* > 500. Since flanked STRs are known to cause problems for correct scaffold orientation during *de novo* genome assembly, the orientation of this scaffold on pseudomolecule 5B of CS (IWGSC assembly) until the appearance of version 1.2 was questionable. The direction of this genomic region in common wheat cultivars, such as *T. aestivum* varieties Arina, Jagger, Julius, and Landmark, is similar to that in Zv (Walkowiak et al., 2020). However, in all of these cases, the scaffolds break at the flanked satDNA. On the other hand, this inversion is absent in the 5B pseudomolecule of the CS42 genome assembly (Zimin et al., 2017), which, together with optical maps, was used to improve the IWGSC CS assembly during the release of RefSeq v2.1 (Zhu et al., 2021). Although these data most likely indicate the absence of an inversion within HDR2, we cannot rule out its presence on chromosomes 5B of CS and CS-5Bdic used in the cross in the present study. Inversion of a scaffold would be a good explanation for the recombination suppression on the 5BL_RS region, although we do not yet have enough evidence to confirm this assumption.

## Discussion

Recombination suppression is an important phenomenon involved in processes such as the formation of sex chromosomes, evolution of hybrid zones, and speciation (Mank, 2012; Charlesworth, 2016). In plant breeding, the transfer of agronomically important genes in non-recombinant linkage groups occurs when they are localized on alien translocations (Wingen et al., 2017). There are diverse causes of recombination suppression; the most commonly discussed are InDels, inversions, heterochromatin regions, and epigenetic mechanisms (Hammarlund et al., 2005; Ziolkowski et al., 2015; Guo et al., 2016).

In our case, recombination-suppression regions on wheat chromosome 5B were detected in a cross between CS and CS-5Bdic (genotype CS with 5B substitution by its homologue from tetraploid *T. dicoccoides*). It is currently not possible to use information on the 5Bdic chromosome donor to establish the possible causes of recombination suppression, although this substitution line has been actively used in various genetic studies (Faris et al., 2000; Lu et al., 2006; Salina et al., 2018). At the same time, much experimental material has been accumulated on intraspecific polymorphism of tetraploid species of the emmer group, which may be useful for identifying the causes of recombination suppression observed in CS × CS-5Bdic crosses. Thus, a comparative chromosome analysis by C-banding in 446 accessions of *T. dicoccum* and 105 accessions of *T. dicoccoides* from seven countries, covering its whole present-day natural distribution range, revealed population-specific and region-specific polymorphisms (Badaeva et al., 2015). The following intraspecific polymorphisms were observed for chromosome 5B in *T. dicoccum* and *T. dicoccoides*: (1) a 7A:5B translocation involving the short arm of chromosome 5B (T5BL.5BS- 7AL); (2) differences in the distribution of diagnostic C-bands on the long chromosome arm. It should be noted that the 5BS_RS and 5BL_RS regions identified in our study also fall into the region of high intraspecific polymorphism. Thus, a more detailed analysis of the T5BL.5BS-7AL breakpoint on chromosome 5BS showed that it is located distal to the minor pSc119.2 site and the 5S rRNA gene locus, whereas the major pSc119.2 site was relocated to chromosome 7A (Dedkova et al., 2009; Badaeva et al., 2015). A comparison of these findings with our data on the location of the recombination-suppression region relative to the distribution of repeats on chromosome 5BS (Supplementary Figure S2) suggests that the region of the chromosome that we labeled 5BS_RS is involved in intraspecific rearrangement in *T. dicoccum* and *T. dicoccoides*. We cannot rule out the possibility that the breakpoint runs in close proximity to the tandem pSc119.2 repeats identified in this region (Figure 2).

Intraspecific polymorphism on the long arm of chromosome 5B was revealed by variation in the location of C bands, the total number of which on 5BL is eight (Badaeva et al., 2015). In *T. dicoccum* accessions, the region attributable to intraspecific polymorphism covers the interstitial and distal region of 5BL, whereas for *T. dicoccoides* accessions, this region is significantly wider and covers more than 70% of 5BL. The 5BL_RS region that we studied, located in the 5BL18 bin (Figure 1), also falls within the region of intraspecific polymorphism of *T. dicoccum* and *T. dicoccoides* species. In addition, as previously shown, alien translocations often occur in the 5BL18 bin of chromosome 5B during interspecific hybridization (Timonova et al., 2013). Taken together, this indicates the relative instability of the regions marked as 5BS_RS and 5BL_RS during both plant adaptation to different agroclimatic zones and interspecific hybridization.

The reasons for such instability, leading to intraspecific differences and subsequently, to the suppression of recombination during interspecific hybridization, can be clarified by a detailed analysis of the structure of the chromosome regions involved in these processes. As possible factors of suppressed recombination in 5BS_RS, 5BL_RS or both, one can consider heterochromatization associated with the presence of extended tracts of tandem repeats (119.2), large insertions, and highly divergent regions enriched with TEs.

### Role of Tandem Repeats and TEs in Recombination Suppression

Tandem repeats and TEs are quite often associated with the heterochromatin regions of chromosomes. The possible participation of heterochromatin regions in recombination suppression has been discussed previously (Charlesworth et al., 1986; Kagawa et al., 2002). There is evidence that heterochromatin suppression of crossing-over is controlled in the chromatin structure (Westphal and Reuter, 2002).

The region of recombination suppression on the short arm of chromosome 5B is colocalized with a heterochromatin region at the boundary of two distal bins (5BS6–5BS5) according to physical and genetic mapping (Figure 1; Salina et al., 2018). Analysis of 5BS_RS revealed that nearly 2.8 Mb of this region is covered by pSc119.2 tandem repeats. The other part of the region is covered by TEs and displays very low between-species similarity due to extended InDels.

One of the most common features of satDNA, which is shared among many eukaryotic organisms, is its intrinsic curvature. In previous studies, it has been repeatedly noted that clusters of tandemly repeated nucleotide sequences probably play a key role in DNA stabilization, as well as in protein packaging and higher-order chromatin condensation (Fitzgerald et al., 1994; Tolstorukov et al., 2005; Mrazek, 2010). It is assumed that sequence-directed bends produced by the repeated A-tracts in the satDNA in phase with the helix facilitate the assembly of DNA into nucleosomes and therefore, represent an essential structural basis for heterochromatin condensation (Fitzgerald et al., 1994; Rohs et al., 2009; Segal and Widom, 2009; Struhl and Segal, 2013). The results obtained in the present study agree with the results of Vershinin and Heslop-Harrison (1998) indicating the phasing of nucleosomes along arrays of the pSc119.2 satDNA. Furthermore, the pSc119.2 repeat is known to form a major part of heterochromatin regions in the B genome of wheat (Mukai et al., 1993; Contento et al., 2005). In the present study, we demonstrated that the localization of A-tracts within the pSc119.2 motif leads to sequence-directed bending of the DNA molecule. We suggest that the observed periodicity of the DNA bend can facilitate molecular packing and may be a structural basis for local heterochromatin condensation in this region. The high degree of similarity between different clusters of pSc119.2 found herein supports the existing opinion that there is selective pressure acting on the maintenance of curved DNA (Charlesworth et al., 1994). Bearing in mind the overlapping location of the studied region with the heterochromatin stretch, we argue that large arrays of pSc119.2 satDNA within the 5BS_RS locus can lead to the formation of local islands of interstitial heterochromatin within the euchromatic chromosomal arm, contributing to recombination suppression in this and adjacent regions.

There are also other mechanisms supporting heterochromatin structure. Thus, direct or inverted repeats can lead to the creation of a heterochromatic state (Bennetzen et al., 1994; Dorer and Henikoff, 1994; Tikhonov et al., 1999). The methylation of histone H3mK9 (Jenuwein and Allis, 2001; Martienssen and Colot, 2001; Vermaak et al., 2003), DNA methylation (Martienssen and Colot, 2001; Law and Jacobsen, 2010) and histone variant H2A.W (Yelagandula et al., 2014) play an important function in regulating heterochromatin and act in synergy to maintain transposon silencing. In particular, in the present study it was shown that chromatin of the pSc119.2 repeat-containing regions within 5BS_RS is marked with histone H3K9me2. This last is the signature of repeat-rich constitutive heterochromatin for many eukaryotes (Naumann et al., 2005; Nicetto and Zaret, 2019) and is an evolutionarily conserved, specific mark of nuclear peripheral heterochromatin (Poleshko et al., 2019).

Furthermore, it has been shown that heterochromatin in *Arabidopsis* is determined by TEs and related tandem repeats under the control of the chromatin-remodeling ATPase DDM1 (Lippman et al., 2004). The enrichment of TE sequences in heterochromatin is widespread among higher eukaryotes. In plant genomes, even relatively small retrotransposon blocks are methylated (Bennetzen et al., 1994; SanMiguel et al., 1996; Tikhonov et al., 1999) and can be considered heterochromatic regions. Moreover, small tandem expansions, such as adjacent duplication of a TE from a monomer to a dimer, or an increase in the copy number within a simple sequence repeat, can lead to altered epigenetic status within and around these sequences, and these may also be considered heterochromatin (Dorer and Henikoff, 1994).

In the present study, during the analysis of the LTR–LTR divergence, we found indirect evidence for heterochromatin formation in the 5BS_RS region carrying extended TE arrays. It was shown that local heterochromatin islands, determined by the satDNA and TE arrays, as well as dissimilarity caused by the large InDels (chromosome rearrangements), are likely key determinants of recombination suppression in the 5BS_RS region.

Our results allow suggesting that mobile genetic elements (both DNA transposons and LTR retrotransposons) played a key role in the loss of similarity in the region labeled 5BL_RS. Overall, two major and two minor segments with significant loss of similarity can be recognized within the 5BL_RS region (HDR1–4, Figure 3A). The HDRs are enriched with TEs, but each region is characterized by a different history of integration and movement of LTR retroelements, and a different distribution ratio of DNA transposons and LTR retrotransposons. The results are consistent with recent studies indicating a negative correlation between regional genomic variation in TE density and frequency of recombination. The strength of this correlation varies depending on the TE type; TE insertion polymorphism may be an important factor determining intraspecific variations in recombination rate in the surrounding genomic regions, with all of the consequences of this event (Kent et al., 2017).

## Conclusion

Chromosomal rearrangements that lead to recombination suppression can have a significant impact on speciation, and they are also important for breeding. In our case, recombination-suppression regions on wheat chromosome 5B (5BS_RS and 5BL_RS) were detected in a cross between CS and CS-5Bdic (genotype CS with 5B substitution by its homologue from tetraploid *T. dicoccoides)*. It was shown that the loss of similarity, which can lead to suppression of recombination in the studied regions, is caused by chromosomal rearrangements, driven by the activity of mobile genetic elements (both DNA transposons and long terminal repeat retrotransposons) and their divergence during evolution. Also, the local heterochromatin islands determined by the satellite DNA (pSc119.2), as well as the dissimilarity caused by large insertions/deletions (chromosome rearrangements) between *aestivum/dicoccoides*, are likely the key determinants of recombination suppression in the region.

It was noted that the regions marked as 5BS_RS and 5BL_RS are associated with chromosomal rearrangements identified earlier by С-banding analysis of intraspecific polymorphism of tetraploid emmer wheat. Taken together, this indicates the relative instability of the studied regions during both plant adaptation to different agroclimatic zones and interspecific hybridization.

## Data Availability Statement

The datasets presented in this study can be found in online repositories. The names of the repository/repositories and accession number(s) can be found in the article/Supplementary Material.

## Author Contributions

ES, AM, and AKo: conceptualization. AKi: formal analysis and investigation (comparative mapping data). ZL: formal analysis and investigation (Langdon × Hermon RIL mapping data). AM: formal analysis and investigation (RS regions of pseudomolecules, satDNA, TEs, large InDels, and DNA shape analyses). ES: project administration and supervision. ES, AM, and AKi: writing-original draft preparation. ES, AM, ZL, and AKo: writing, review, and editing. All authors contributed to the article and approved the submitted version.

## Funding

Genetic linkage maps and comparative chromosome analysis were supported by RSF (Russian Science Foundation) project no. 21-76-30003. Analysis of the 5BS_RS and 5BL-RS regions was funded by the Kurchatov Genomics Center of IC&G (075-15-2019-1662). DNA shape analysis was performed within the budgetary project FWNR-2022-0017.

## Conflict of Interest

The authors declare that the research was conducted in the absence of any commercial or financial relationships that could be construed as a potential conflict of interest.

## Publisher’s Note

All claims expressed in this article are solely those of the authors and do not necessarily represent those of their affiliated organizations, or those of the publisher, the editors and the reviewers. Any product that may be evaluated in this article, or claim that may be made by its manufacturer, is not guaranteed or endorsed by the publisher.

## Footnotes

^1^ https://wheat-urgi.versailles.inra.fr/Seq-Repository

^2^ http://wewseq.wixsite.com/consortium

^3^ ftp://ftp.ncbi.nlm.nih.gov/genomes

^4^ http://webblast.ipk-gatersleben.de/wheat_ten_genomes

## Supplementary Material

Supplementary Figure S1 **(A)** Scatter graph of distribution of number of pSc119.2 clusters with length not less than 1 kb, within the 5BS_RS region extracted from Cs and Zv pseudomolecules and divided on 100-kb segment. **(B)** Scatter graph of coverage by the sequences of the pSc119.2 clusters evaluated for each 100-kb segment. Arrows indicate direction (forward/inverse) of the pcS119.2 satDNA arrays.

Supplementary Figure S2 Distribution of the pSc119.2 arrays on pseudomolecule of 5BS. Localization of telomere, centromere, and the recombination suppression region are indicated. Forward and reverse orientation of pSc119.2 is indicated by the blue and red colors, respectively. The pSc119.2 was mapped and plotted in MUMmer 3.0 (Kurtz et al., 2004).

Supplementary Figure S3 Distribution of the E12 and E13 chromatin states within 5BS_RS and their signatures (Li et al., 2019).

Supplementary Table S2 Markers flanking regions of suppression. The markers flanking 5BS_RS and 5BL_RS are in red.
