# Supplementary figures and images for: Dissection of Structural Reorganization of Wheat 5B Chromosome Associated With Interspecies Recombination Suppression
Source: Front Plant Sci. 2022 May 4;13:884632. doi: 10.3389/fpls.2022.884632 (PMC9629394; doi:10.3389/fpls.2022.884632)

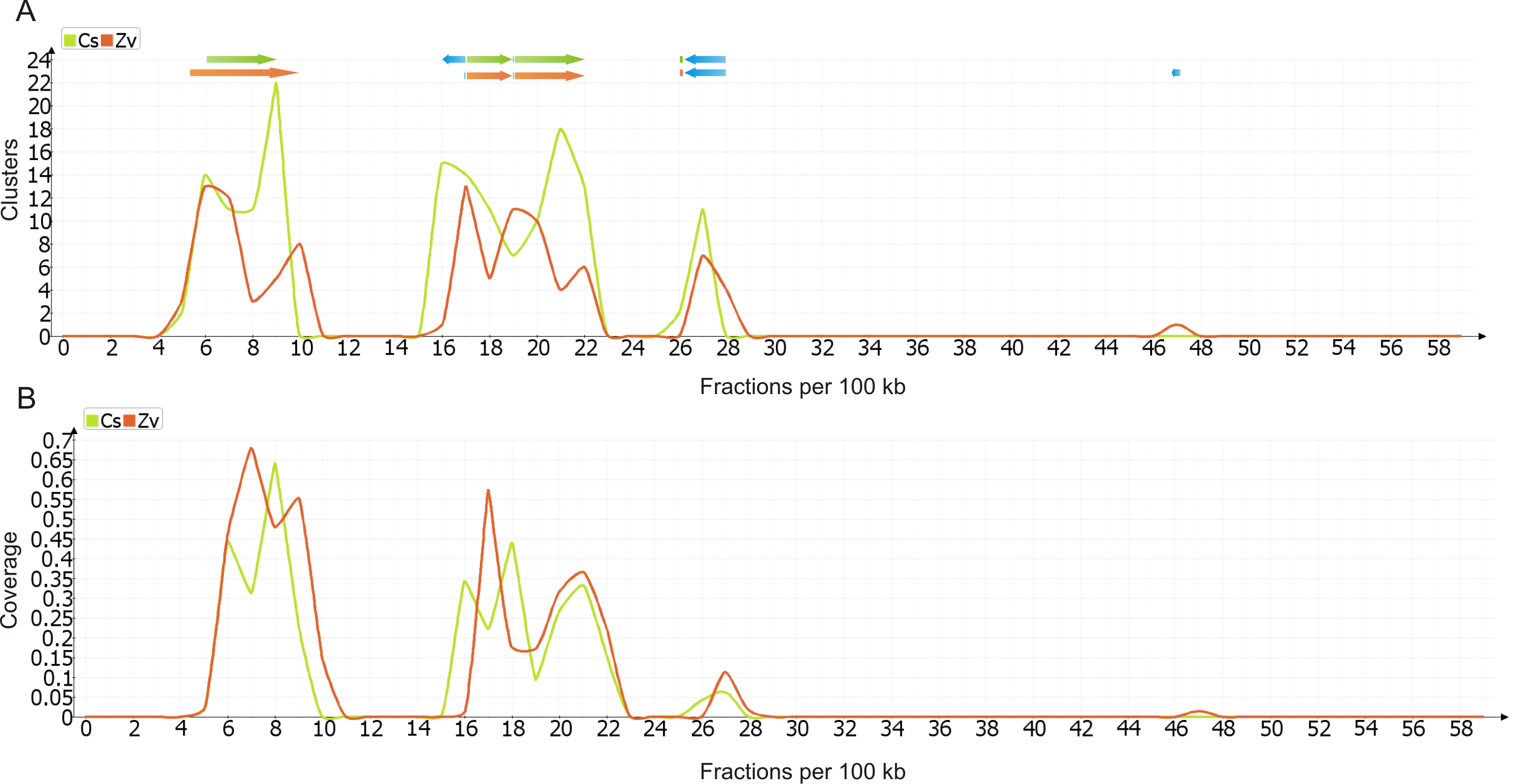

Supplement: Supplementary Figure S1 — (A) Scatter graph of distribution of number of pSc119.2 clusters with length not less than 1 kb, within the 5BS_RS region extracted from Cs and Zv pseudomolecules and divided on 100-kb segment. (B) Scatter graph of coverage by the sequences of the pSc119.2 clusters evaluated for each 100-kb segment. Arrows indicate direction (forward/inverse) of the pcS119.2 satDNA arrays. [file Image_1.TIF]

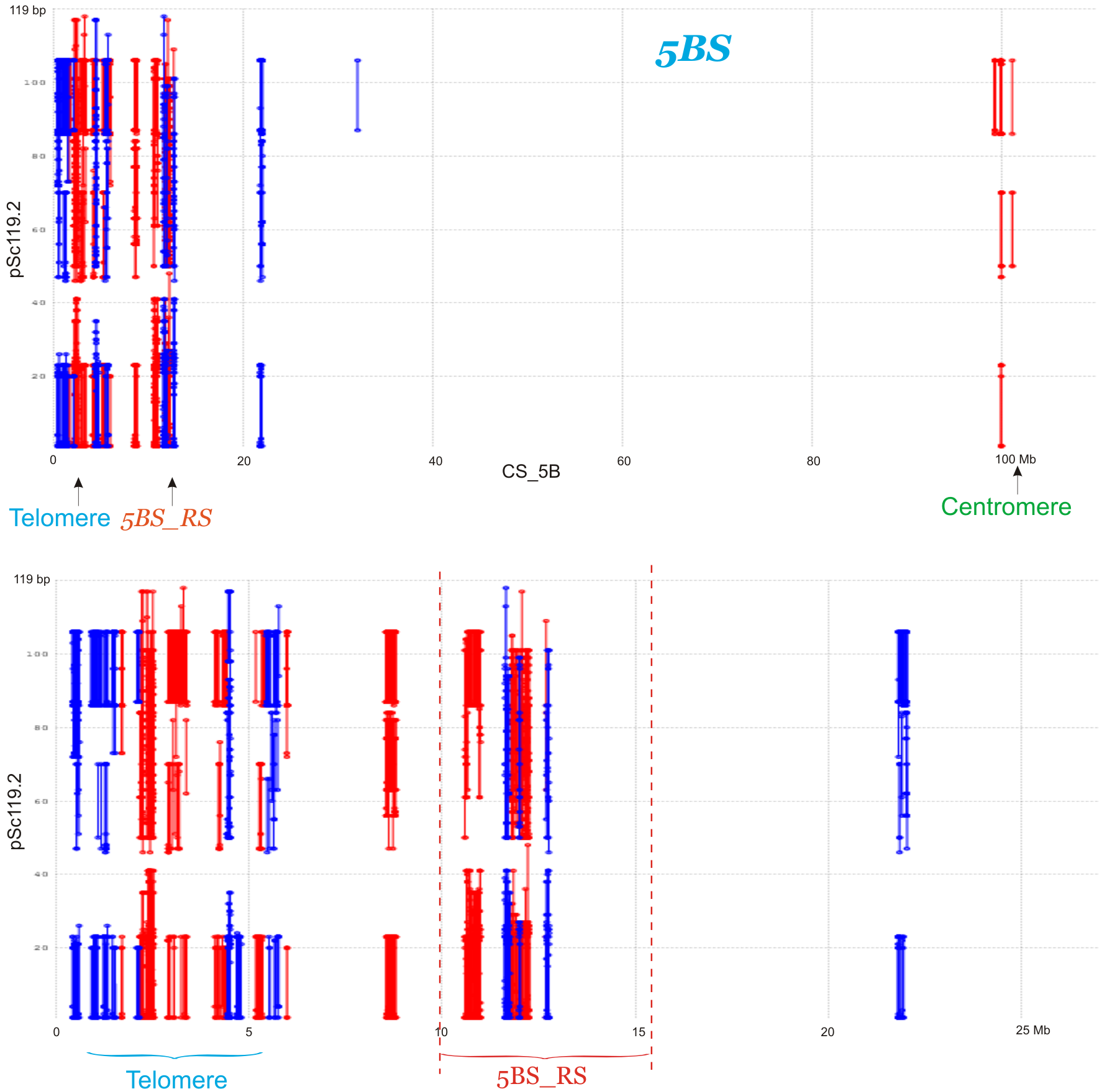

Supplement: Supplementary Figure S2 — Distribution of the pSc119.2 arrays on pseudomolecule of 5BS. Localization of telomere, centromere, and the recombination suppression region are indicated. Forward and reverse orientation of pSc119.2 is indicated by the blue and red colors, respectively. The pSc119.2 was mapped and plotted in MUMmer 3.0 (Kurtz et al., 2004). [file Image_2.TIF]

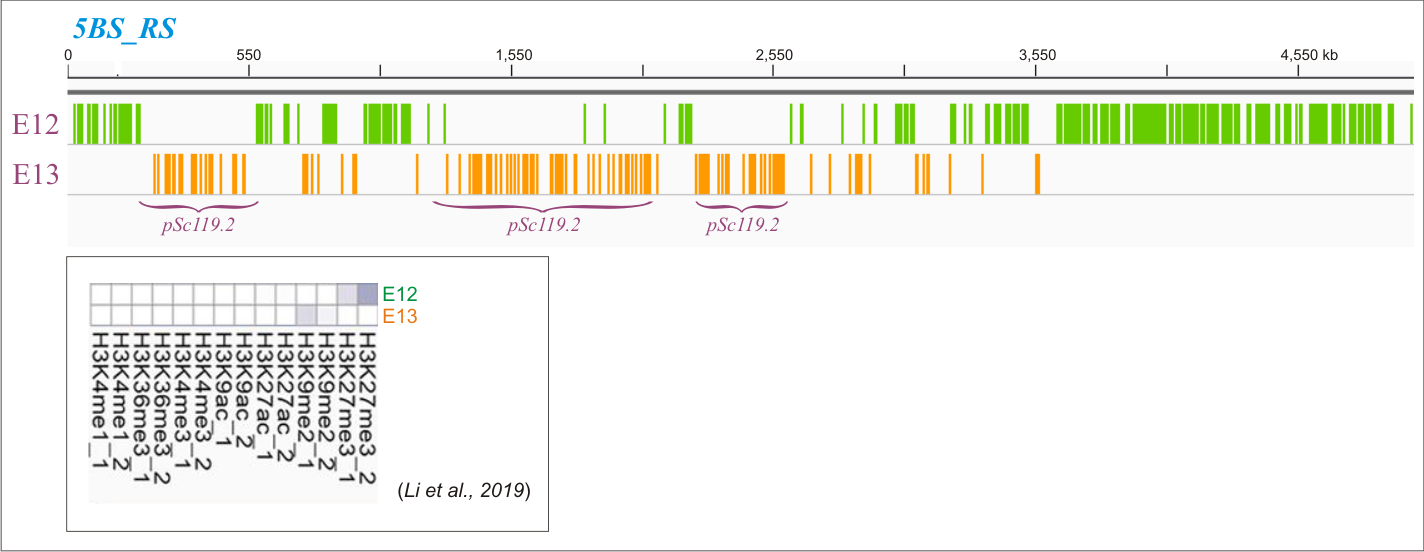

Supplement: Supplementary Figure S3 — Distribution of the E12 and E13 chromatin states within 5BS_RS and their signatures (Li et al., 2019). [file Image_3.TIF]
