# Supplementary material for: Dissection of Structural Reorganization of Wheat 5B Chromosome Associated With Interspecies Recombination Suppression
Source: Front Plant Sci. 2022 May 4;13:884632. doi: 10.3389/fpls.2022.884632 (PMC9629394; doi:10.3389/fpls.2022.884632)
Supplement: Supplementary file 4 [file Table_1.DOCX]

Table 1S. Details of mapping populations used in this study.

| Parent varieties | Chinese Spring x Chinese Spring-5Bdic | Chara x Glenlea | Langdon x Hermon | Svevo x Zavitan |
| --- | --- | --- | --- | --- |
| Acronym | CS x CS-5Bdic | Cha x Glen | Ldn x Hr | Sv x Zv |
| Parent species | *T. aestivum* x *T*. *dicoccoides* | *T. aestivum* x *T. aestivum* | *T. durum x T. dicoccoides* | *T. durum* x *T. dicoccoides* |
| Population type | RIL | DH | RIL | RIL |
| Array | Illumina Infinium 15K | Illumina Infinium 90K | Illumina Infinium 15K | Illumina Infinium 90K |
| Total number of markers on 5B chromosome | 379 | 1264 | 393 | 1156 |
| Skeletal markers on 5B chromosome | 91 | 83 | 146 | 180 |
| Source | Published in Salina et al., 2018 | Genotyping data provided by E. Akhunov | Personal communication with A. Korol | Published in (Avni et al. 2014) |
